# A novel process for biodiesel production from sludge palm oil

**DOI:** 10.1016/j.mex.2019.09.039

**Published:** 2019-10-18

**Authors:** Papasanee Muanruksa, James Winterburn, Pakawadee Kaewkannetra

**Affiliations:** a Graduate School of Khon Kaen University, Khon Kaen, 40002, Thailand; b Department of Chemical Engineering and Analytical Sciences (CEAS), The University of Manchester, Manchester, M13 9PL, United Kingdom; c Centre for Alternative Energy Research and Development (AERD), Faculty of Engineering, Khon Kaen University, Khon Kaen, 40002, Thailand; d Department of Biotechnology, Faculty of Technology, Khon Kaen University, Khon Kaen, 40002, Thailand

**Keywords:** Fatty acid extraction and enzymatic esterification, Sludge palm oil (SPO), Fatty acids extraction, Esterification, Immobilised alginate-PVA lipase bead, Biodiesel

## Abstract

Typically, sludge palm oil (SPO) which discharged from the palm oil refining process, is a low cost material of potential value, due to its high free fatty acid content. Accordingly, there is potential for upgrading low grade oil into valuable biofuel. In this work, we present a novel method for biodiesel production from SPO. The process consists of two steps (i) free fatty acid (FFA) extraction (ii) enzymatic esterification. Firstly, SPO was saponified by hydroalcoholic solution into soap and glycerol. Secondly, the FFAs obtained were further converted into biodiesel via enzymatic esterification catalyzed by immobilised alginate-PVA lipase beads.

• *Biodiesel production from sludge palm oil could be completed.*
• *A modified fatty acid extraction was used for SPO fatty acid preparation.*
• *Immobilised alginate-PVA lipase beads were used as biocatalyst for esterification reaction.*

## Graphical abstract

**Figure:**
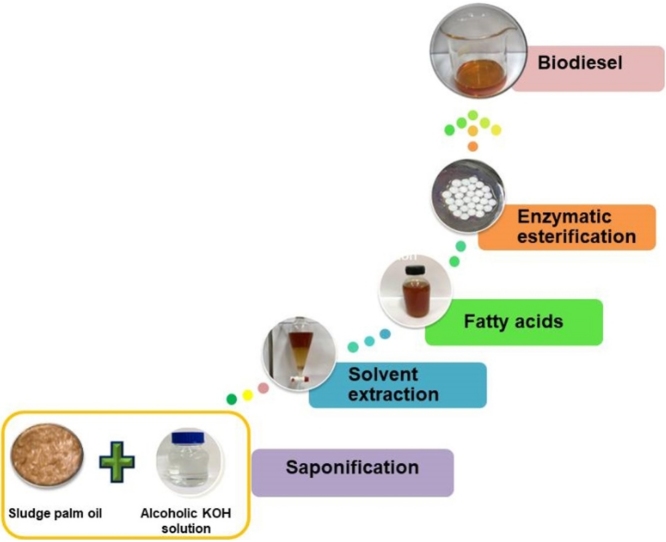

**Specification Table**

**Table:** 

Subject Area:	Energy
More specific subject area:	Biofuel of Biodiesel
Method name:	Fatty acid extraction and enzymatic esterification
Name and reference of original method:	Production of biodiesel from vegetable oil and microalgae by fatty acid extraction and enzymatic esterification.
Resource availability:	DOI: https://doi.org/10.1016/j.jbiosc.2014.11.002

## Method details

Generally, palm oil refinery processing plants release waste water streams which are known as palm oil mill effluent (POME). The main residue oil found in the upper layer of POME is sludge palm oil (SPO). SPO can be separated from the first stage of POME discharge [1]. SPO is classified as low grade oil due to its high free fatty acid (FFA) content (>20%) [2]. The FFA in SPO can be used as supplements in animal feed, low-grade soap and as feedstock for biogas production [[3], [4], [5], [6]]. Furthermore, SPO is considered as a low cost substrate (1 USD/Ton) for biodiesel production. Conversion of SPO to biodiesel is an effective means of waste utilisation and provides a low cost biodiesel production route. Normally, biodiesel is produced via a transesterification reaction using a homogenous alkaline catalyst. This process has several advantages such as short reaction time, high conversion and low catalyst use [7]. However, the FFA and water content in low grade oil causes soap formation during transesterification reaction catalysed by homogeneous alkaline catalyst. On the other hand, homogenous acid catalysts are not affected by the presence of FFA, but conversion of FFAs to biodiesel using acid catalyst is also hindered by aspects such as low reaction rate, long reaction time, corrosive solvents used and low biodiesel yield [8]. For heterogeneous acidic and basic catalysts, there are many advantages such as low costs, reusability and simultaneous catalysis of transesteriﬁcation and esteriﬁcation reactions. However, heterogeneous catalysis is disadvantageous because it has limitations of active sites and mass transfer resulting in low reaction rates [9,10]. In term of an enzymatic process, transesteriﬁcation of triglyceride and esteriﬁcation of FFA can be promoted simultaneously. However, the high cost of enzymes and deactivation are a major drawback [11,12]. There are many research works which report biodiesel production from SPO using homogeneous alkaline catalysts, homogeneous acid catalysts, heterogeneous acidic and basic catalysts and biocatalysts as shown in Table 1. One previous study by the authors [13] reports the production of biodiesel from SPO via FFA extraction coupled with enzymatic esterification, using immobilised lipase as biocatalyst. The process was developed to solve problems of soap formation, low conversion, long reaction time, reusability and enzyme deactivation. The novel method developed in [13] to produce biodiesel from SPO based on a combination of chemical and biological routes is described in detail here. There are two steps of this process (i) obtaining FFA from SPO via saponification and solvent extraction (ii) conversion of FFA to biodiesel via enzymatic esterification.

**Table 1:** Production of biodiesel from sludge palm oil (SPO) via different processes.

References	Processes and conditions	Biodiesel yield (%)	Reusability of catalyst
[13]	**Fatty acids extraction/ enzymatic esterification** **Fatty acids extraction:** alcoholic KOH solution and SPO (2:1), reaction temperature 60 °C, agitation speed 250 rpm and 30 min. **Enzymatic esterification:** biocatalyst (immobilised *R. oryzae* lipase) 2% wt., reaction temperature 40 °C, methanol to FFA molar ratio 3:1, agitation speed 200 rpm and 4 h.	91.30	15
[14]	**Tranesterification** **Transesterification:** lipase from pacific white shrimp 40 kUnit, reaction temperature 40 °C, methanol to oil molar ratio 6:1, water content 3%, agitation speed 250 rpm, and 12 h.	91.45	–
[15]	**Transesterification** Transesterification: locally-produced *Canida cylindracea* lipase 10U/25 g of SPO, reaction temperature 40 °C, ethanol to SPO molar ratio 4:1, additional of tert-butanol-to-SPO molar ratio 2:1 into the ethanol–solvent system, agitation speed 200 rpm and 24 h.	71.60	–
[16]	**Transesterification** **Transesterification:** crude lipase from oil palm fruit 36 U/ 10 g of oil, reaction temperature 35 °C, methanol to oil ratio 6:1, agitation speed 200 rpm and 36 h.	92.07	–
[17]	**Transesterification** **Transesterification:** NaOH catalyst 1% wt., reaction temperature 70 °C, ethanol to oil molar ratio 9:1, deep eutectic solvent (DES) 4%, agitation speed 400 rpm and 1 h.	83.19	–
[2]	**Esterification/ Transesterification** **Esterification:** alum catalyst 6%, reaction temperature 60 °C, methanol to oil molar ratio 20:1, agitation speed 300 rpm and 3 h. **Transesterification:** KOH catalyst 1.5%, reaction temperature 60 °C, agitation speed 300 rpm and 1 h.	93.00	–
[18]	**Esterification/ Transesterification** **Esterification:** Trifluoromethanessulfonic (TFMSA) catalyst 0.75%, reaction temperature 60 °C, methanol to oil molar ratio 10:1, agitation speed 300 rpm and 30 min. **Transesterification:** KOH catalyst 1%, reaction temperature 60 °C, agitation speed 300 rpm and 60 min.	84.00	–
[19]	**Transesterification** **Transesterification:***Canida cylindracea* lipase 10 U, reaction temperature 40 °C, ethanol to oil ratio 4:1, agitation speed 250 rpm and 24 h.	62.30	–
[20]	**Esterification/ Transesterification** **Esterification:** H_2_SO_4_ catalyst 0.75%, reaction temperature 60 °C, methanol to oil molar ratio 8:1, agitation speed 400 rpm and 60 min. **Transesterification:** KOH catalyst 1%, reaction temperature 60 °C, methanol to oil molar ratio 10:1, agitation speed 400 rpm and 60 min.	83.72	–
[21]	**Esterification/ Transesterification** **Esterification:** P-toluene-4-sulfonic monohydrate acid (PTSA) catalyst 0.75% wt., reaction temperature 60 °C, methanol to oil molar ratio 10:1, agitation speed 400 rpm and 60 min. **Transesterification:** KOH catalyst 1%, reaction temperature 60 °C, methanol to oil molar ratio 10:1, agitation speed 400 rpm and 60 min.	76.62	–
[22]	**Esterification** **Esterification:** P-toluene-4-sulfonic monohydrate acid (PTSA) catalyst 0.75% wt., reaction temperature 60 °C, methanol to oil molar ratio 10:1, agitation speed 400 rpm and 60 min.	96.00	–

## Materials

1 Sludge palm oil
2 Cotton sheet
3 Bovine serum albumin
4 Lipase from *Rhizopus oryzae*
5 Olive oil (Food grade)
6 Alginate (Food grade)
7 Potassium Hydroxide (Analytical grade)
8 Ethanol (95–99%) (Analytical grade)
9 Methanol (95–99%) (Analytical grade)
10 Phenolphthalein (Analytical grade)
11 Hydrochloric acid 37% (Analytical grade)
12 Calcium chloride (Analytical grade)
13 Sodium hydroxide (Analytical grade)
14 Monobasic sodium phosphate
15 Copper sulphate (Analytical grade)
16 Polyvinyl alcohol (Analytical grade)

## Pretreatment of sludge palm oil

Samples of sludge palm oil (SPO) were collected from Esarn palm oil (ESP) company, Sakon Nakhon province, North-eastern Thailand. The SPO was heated to 80 °C and filtered through cotton sheet prior to use.

## Fatty acid extraction

**Fig. 1:**
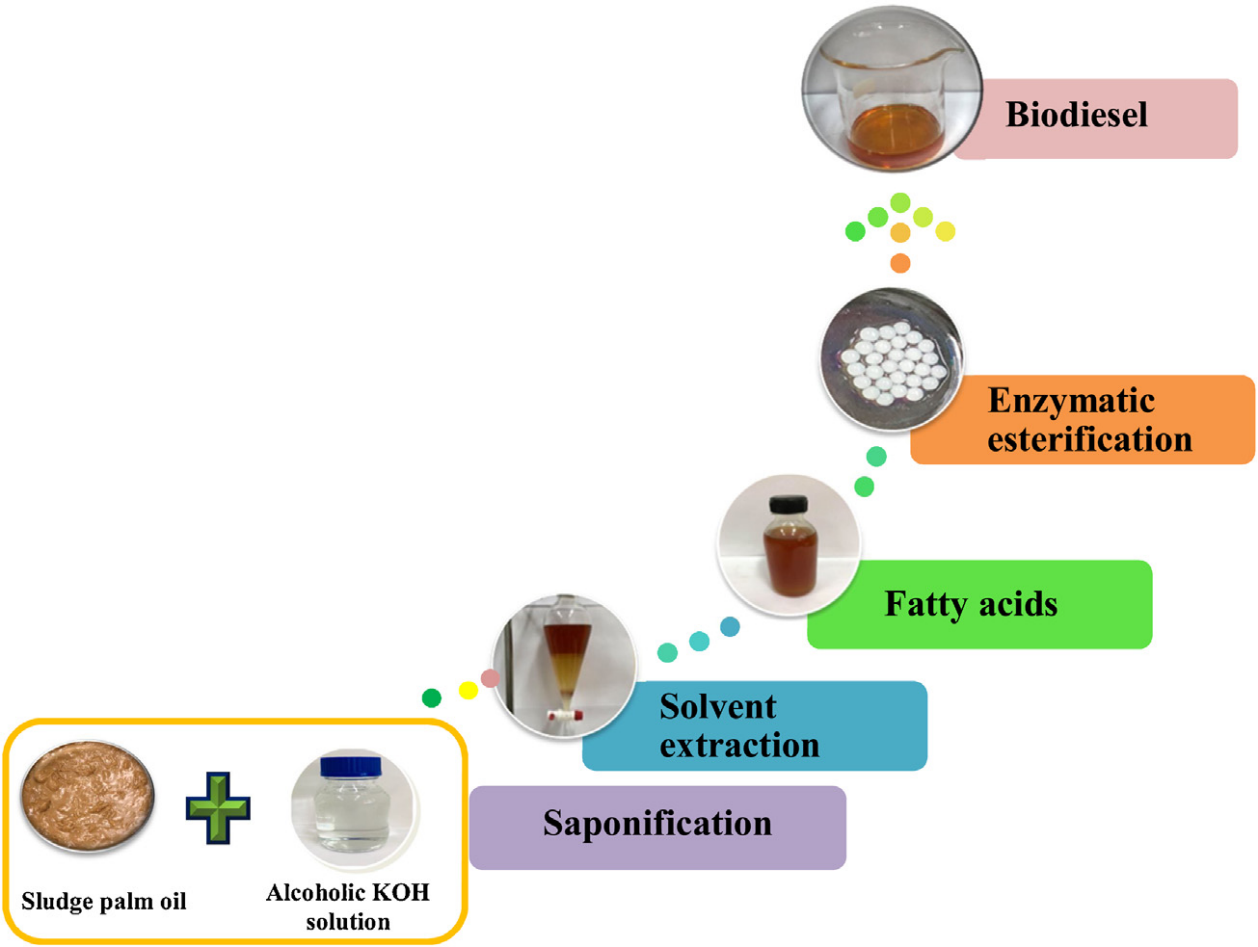
Flow diagram of biodiesel production from sludge palm oil via combination of fatty acids extraction and enzymatic esterification.

Free fatty acid (FFA) from SPO was obtained by direct saponification according to a modified method explained in a previous study [23]. FFA extraction was carried out using hydroalcoholic solution, which was prepared which was prepared dissolving 120 g of NaOH in 400 mL of water and 400 mL of ethanol. In this work, there were three main steps for FFA extraction as shown in Fig. 1. Firstly, 5 M alcoholic KOH solution was prepared by dissolving 280 g of potassium hydroxide (KOH) in 1000 mL ethanol, after which 50 mL SPO was mixed with alcoholic KOH solution at a ratio of 1:2. The saponification was conducted by shaking the mixture at 250 rpm, 60 °C for 30 min. The reaction mixture was then cooled down to room temperature by adding water (45 mL) and pH was adjusted to 1 using 37% HCl to form FFA. The FFA were extracted with hexane (45 mL) at 250 rpm, 30 °C for 30 min. The hydroalcoholic and hexane phases were subsequently separated using a separating funnel and the FFA rich hexane phase was washed twice with water. Finally, hexane was evaporated and the resulting FFA solution was washed 3 times with water prior to use in further experiments.

## Lipase immobilisation

A solution of lipase (5% w/v) in phosphate buffer 10 mL (pH 7.0) was mixed with 90 mL of matrix solution between alginate and polyvinyl alcohol (ratio of 1:1). To form the immobilised beads, the mixture solution was added dropwise into 0.1 M CaCl_2_ by sterile syringe and was kept at 4 °C for 24 h. Lastly, the alginate-PVA beads were washed with distilled water three times and stored at 4 °C for use in further experiments.

## Esterification reaction

Esterification of FFA from SPO was performed in a 250 mL Erlenmeyer flask containing of 50 mL FFA, methanol and biocatalyst (2% w/v). The reaction was optimised by temperature (30 to 50 °C), methanol to fatty acid molar ratio (1:1 to 5:1), and agitation speed (100 to 300 rpm). The analytical procedure is described in Section 7.4.

## Analytical techniques

### Determination of lipase activity

Lipase activity was determined by the olive oil emulsion method [24]. Immobilised lipase was mixed with a solution of olive oil and PVA (4% w/v) in ratio of 1:3 and incubated at 37 °C for 30 min. The reaction was stopped by adding a solution of acetone and ethanol (1:1 ratio). Finally, a sample was taken to analyse FFA content (7.3). One unit of lipase activity was defined as the release of 1 μmol oleic acid per minute under the assay conditions. Specific activity was defined as the number of enzyme units per milligram protein or milligram support.

### Determination of immobilised yield (%)

Immobilised yield was defined as reduction of protein concentration in solution following the method described by Li et al [24]. Protein in solution was measured by Lowry’s method and the immobilisation yield can be calculated as shown in Eq. (1).

(1) $$\text{Immobilisation}\text{ yield =}\frac{\text{C}\text{i}-\text{C}\text{f}}{\text{C}\text{i}}\text{×100\%}$$

where *C_i_* = initial protein concentration without matrix support (mg/ml); *C_f_* = protein concentration after immobilisation (mg/ml)

### Determination of FFA content (%)

The percentage of FFA in the oil sample was determined according to an official method of the American Oil Chemistry Society (Ca 5a-40). Firstly, 0.1 g of oil sample was dissolved in ethanol and the mixture solution was then titrated with NaOH (0.05 M) using phenolphthalein to indicate the endpoint, with the appearance and persistence of a pink colour indicating endpoint of the titration. FFA content (%) can be calculated as shown in Eq. (2).

(2) $$\text{FFA content (\%)}\;=\;\frac{\text{N}\times \text{V}\times \text{MW}}{\text{10 ×}\text{g}}$$

where *N* = normality of NaOH; *V* = volume of NaOH consumed (mL); *MW* = average molecular weight of fatty acids in oil sample (g/mol); *g* = sample weight (g)

### Determination of esterification degree (%)

The degree of esterification (%) was determined as the reduction of FFA content in oil sample [20]. The degree of the reaction can be calculated using Eq. (3).

(3) $$\text{Esterification degree }\left(\text{\%}\right)\text{= }\frac{\text{A}\text{i}-\text{A}\text{f}}{\text{A}\text{i}}\text{ ×100}$$

where *A_i_* = free fatty acid content in sample at initial time; *A_f_* = free fatty acid content in sample after the reaction

### Analysis of biodiesel composition

The FAME product was evaluated following the European Standard method (EN 14103:2003). Firstly, 250 mg sample was filled into a 10 mL vial followed by the addition of 5 mL of internal standard (methyl haptadecanoate solution 10 mg/ml). Subsequently, the sample was analysed by Gas Chromatography – Mass Spectroscopy (GC–MS) (GC-2010, Shimadzu, Japan) equipped with a 30 m, long and 0.25 mm diameter capillary column, lined with a 0.25 μm (Rtx-5 ms, Rextex). Samples were injected in split/column flow ratio 24:1. Helium was used as the carrier gas, flow rate 1 ml/min. The injection temperature was 250 °C and column oven 250 °C (programmed to start at 120 °C, held at this temperature for 5 min and heated at a rate of 3 °C/min to 250 °C).

### Statistical analysis

All experiments were carried out in triplicate as a minimum for reproducibility and the data obtained was reported as mean ± SD. The data were analysed using single factor ANOVA with Microsoft Excel 2010. The results were considered statistically significant for P value less than 0.05.
